# Current Management and Future Perspectives in the Treatment of Lp(a) with a Focus on the Prevention of Cardiovascular Diseases

**DOI:** 10.3390/ph16070919

**Published:** 2023-06-23

**Authors:** Juan M. Farina, Milagros Pereyra, Ahmed K. Mahmoud, Chieh-Ju Chao, Timothy Barry, Susan M. Halli Demeter, Chadi Ayoub, Reza Arsanjani

**Affiliations:** 1 Department of Cardiovascular Medicine, Mayo Clinic, 5777 E Mayo Blvd, Phoenix, AZ 85054, USA; 2 Department of Cardiovascular Medicine, Mayo Clinic, Rochester, MN 55905, USA

**Keywords:** lipoprotein(a), cardiovascular diseases, treatment, RNA interference

## Abstract

Lipoprotein(a) [Lp(a)] is a lipid molecule with atherogenic, inflammatory, thrombotic, and antifibrinolytic effects, whose concentrations are predominantly genetically determined. The association between Lp(a) and cardiovascular diseases (CVDs) has been well-established in numerous studies, and the ability to measure Lp(a) levels is widely available in the community. As such, there has been increasing interest in Lp(a) as a therapeutic target for the prevention of CVD. The impact of the currently available lipid-modifying agents on Lp(a) is modest and heterogeneous, except for the monoclonal antibody proprotein convertase subtilisin/kexin type 9 inhibitors (PCSK9i), which demonstrated a significant reduction in Lp(a) levels. However, the absolute reduction in Lp(a) to significantly decrease CVD outcomes has not been definitely established, and the magnitude of the effect of PCSK9i seems insufficient to directly reduce the Lp(a)-related CVD risk. Therefore, emerging therapies are being developed that specifically aim to lower Lp(a) levels and the risk of CVD, including RNA interference (RNAi) agents, which have the capacity for temporary and reversible downregulation of gene expression. This review article aims to summarize the effects of Lp(a) on CVD and to evaluate the available evidence on established and emerging therapies targeting Lp(a) levels, focusing on the potential reduction of CVD risk attributable to Lp(a) concentrations.

## 1. Introduction

HMG-CoA reductase inhibitors (statins); other lipid-modifying agents, such as ezetimibe; and proprotein convertase subtilisin/kexin type 9 inhibitors (PCSK9i) have demonstrated clinical benefits for reducing the risk of cardiovascular diseases (CVDs), mainly through lowering circulating levels of low-density lipoprotein (LDL) cholesterol [1,2,3,4]. However, despite the effective control of LDL and other known modifiable risk factors, for some patients, a residual risk of major cardiovascular events (MACEs) remains [5,6,7]. Consequently, further investigation into unidentified CVD risk factors has become increasingly important.

Lipoprotein(a) [Lp(a)], a form of LDL, has emerged as an important and strong risk factor for CVD. First identified in the 1960s, the most robust evidence of its causal role in the risk of CVD has been generated in the last decade [8]. Elevated levels of Lp(a) are well recognized as an independent and causal risk factor for CVD through mechanisms associated with atherogenesis, inflammation, and thrombosis. International guidelines for CVD prevention have recently incorporated Lp(a) as a risk enhancer for elevated LDL [9,10,11]. It is estimated that up to 20% of the world’s population has levels of Lp(a) that result in an increased risk of CVD [12,13].

Lp(a) is predominantly a monogenic cardiovascular risk determinant [14]. Unlike other lipoproteins, for which diet, lifestyle, and genetics play a substantial role in determining plasma levels, Lp(a) concentrations are more than 90% genetically determined by constitutive hepatocyte production [15,16]. However, several studies conducted in pediatric and adult patients have shown that Lp(a) levels increase with older age, thus challenging the assumption that definitive Lp(a) levels are reached at an early age and do not change during adulthood [17,18]. Elevations in Lp(a) levels may be also observed during pregnancy, during menopause, during stress, and with infections [18,19]. Ethnicity may also affect Lp(a) concentrations, with concentrations being higher in Chinese, South Asian, and Black individuals. However, sample sizes and variability in research conducted pose limitations on the generalizability of these findings [20]. Furthermore, sex could also influence Lp(a) levels, with women having 5–10% higher concentrations than men [21].

Historically, there has been relatively little attention given in the clinical setting to the role of Lp(a) as a cardiovascular risk factor. There have been several reasons contributing to this. Firstly, the LPA gene, which is responsible for the production of apolipoprotein A [apo(a)], is not present in lower mammals or prosimians, thus limiting its study in animal models [8]. Additionally, early attempts to lower the CVD risk related to Lp(a) levels by using statins, which are ineffective in directly reducing Lp(a) levels, or other lipid-lowering agents which have modest effects on Lp(a), did not show any improvement in cardiovascular outcomes [8]. Therefore, although effective interventions are now available for many other lipid-related cardiovascular risk factors, Lp(a) has remained an effectively untreatable dyslipidemia. Consequently, emerging therapies are being developed to specifically lower Lp(a) levels and the associated risk of CVD, including RNA interference (RNAi) agents, which have the capacity for temporary and reversible downregulation of gene expression.

This review article aims to summarize the effects of Lp(a) on CVD and to evaluate the available evidence on established and emerging therapies targeting Lp(a) levels, focusing on the potential reduction of CVD risk attributable to Lp(a) concentrations.

## 2. Biochemistry of Lp(a)

Lp(a) is an LDL-like molecule that contains two domains: the apolipoprotein B-100 (apoB), which is encoded by the APOB gene and binds to hydrophilic highly glycosylated apo(a), which, in turn, is encoded by the LPA gene [22,23]. The apo(a) portion determines the differences in density and electrophoretic mobility between LDL and Lp(a) [24], and it was formerly termed “Lp(a) antigen” [25]. Apo(a) has key associations with Lp(a), as this plasminogen (PLG)-like particle imparts unique synthetic, catabolic, and functional characteristics [23]. Apo(a) and PLG share high amino acid sequence similarities in several regions, including the protease domain and the loop-like structures called “Kringles” [23]. Unlike PLG, apo(a) contains two kringles domains (KIV and KV) instead of five [26]. Each kringle contains six conserved cysteine residues that form three disulfide bonds to provide the characteristic triple-loop structure [26]. These loop-like structures are also present in other coagulation factors, such as prothrombin, urokinase, and tissue-type PLG activators [15]. 

Plasma Lp(a) arises from the codominant expression of two LPA gene alleles [27]. The KIV copy number variant is inversely proportional to the Lp(a) concentration, and it is responsible for heterogenicity in the Lp(a) concentration between different individuals [15]. Additionally, numerous single-nucleotide polymorphisms (SNPs) in the LPA locus are strongly associated with Lp(a) levels [28].

## 3. Association between Lp(a) and Cardiovascular Diseases

**Figure 1:**
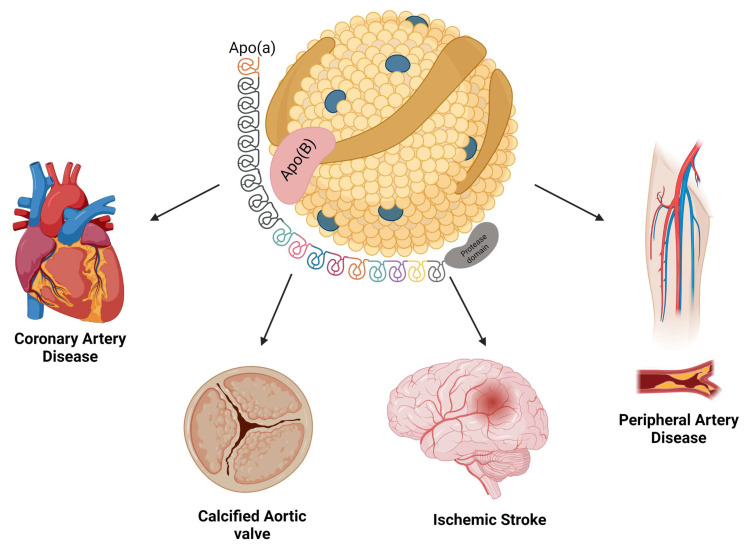
The relationship between Lp(a) and cardiovascular diseases has been well-established in several studies across the last decades, with robust evidence linking elevated Lp(a) levels with atherosclerotic cardiovascular disease, coronary artery disease, cerebrovascular disease, calcific aortic valve stenosis, aortic dissection, peripheral arterial disease, and heart failure.

The relationship between Lp(a) and cardiovascular events has been well-established in several studies across the last decades, and Lp(a) is currently considered the strongest single genetic risk factor for CVD [29]. Collectively, there is robust evidence linking elevated Lp(a) levels with relevant cardiovascular endpoints, such as atherosclerotic cardiovascular disease (ASCVD), coronary artery disease (CAD), cerebrovascular disease, calcific aortic valve stenosis (AS), aortic dissection (AD), peripheral arterial disease (PAD), and heart failure (HF) (Figure 1). A relationship between type 2 diabetes mellitus (DM) and thrombosis, considered a potential risk factor for the development of many cardiovascular conditions, has also been described [21,30,31,32,33,34,35,36].

### 3.1. Atherosclerosis and Coronary Artery Disease

Lp(a) promotes atherosclerotic plaque formation through various mechanisms: it induces the expression of inflammatory cytokines, increases the expression of adhesion molecules on the surface of the endothelial cells, promotes monocyte chemotaxis, and binds transport oxidized phospholipids (OxPLs), which are involved in plaque vulnerability and destabilization [37]. 

A strong association between high serum levels of Lp(a) and atherosclerosis has been reported by several large prospective population-based studies, with the most recent ones analyzing the connection between high serum Lp(a) levels and high baseline atherosclerotic plaque volumes and the presence of adverse plaque features in patients with CAD [10,38]. A post hoc analysis of six randomized controlled trials (RCTs) employing intravascular ultrasound demonstrated that elevated Lp(a) is associated with increased coronary atheroma volume, a finding that supports a pro-atherogenic mechanism of cardiovascular risk related to Lp(a) [39]. Kaiser et al. investigated the relationship between Lp(a) and plaque progression by coronary computed tomography in 191 patients with advanced stable CAD who were already using guideline-directed preventive therapies [40]. After a 12-month follow-up, Lp(a) was associated with accelerated progression of coronary low-attenuation plaque (necrotic core), which may explain the association between Lp(a) and the high residual risk of myocardial infarction. In another study conducted by Mehta et al., plasma Lp(a) and coronary artery calcium (CAC) score were measured at enrollment among asymptomatic participants of the MESA (Multi-Ethnic Study of Atherosclerosis) (n = 4512) and DHS (Dallas Heart Study) (n = 2078) studies [41]. On long-term follow-up (13.2 years in MESA and 11.0 years in DHS), the Lp(a) and CAC score were independently associated with ASCVD risk among participants. The presence of elevated levels of both markers identified a subgroup with a significantly increased ASCVD risk who may benefit from more aggressive therapeutic strategies. 

### 3.2. Cerebrovascular Disease

The role of Lp(a) as a risk factor for stroke is less well documented than for CAD, and large population-based cohort studies on stroke have produced heterogeneous results [42,43]. However, a recent systematic review and meta-analysis including 41 studies with 7874 ischemic stroke patients and 7 studies with 871 intracerebral hemorrhage cases demonstrated a significant association between increased levels of Lp(a) and risk of ischemic stroke as compared to control subjects [44]. Lp(a) levels were also found to be significantly associated with the risk of large artery atherosclerosis subtype of ischemic stroke, as well as being significantly associated with the risk of intracerebral hemorrhage.

### 3.3. Calcific Aortic Valve Stenosis

The identification and better understanding of risk factors for AS is likely to help medical and scientific communities develop novel treatment strategies. A large prospective study by Arsenault et al. was among the first to demonstrate the association between high Lp(a) levels and the risk of AS [31]. They found that a common genetic variant (rs10455872) in the LPA locus was simultaneously associated with both serum Lp(a) levels and the risk of AS, further suggesting that this association was likely to be causal [31]. 

More recent studies have demonstrated that, among AS patients, cases with elevated Lp(a) and OxPL plasma levels exhibit increased valvular calcification activity, faster disease progression, and increased risk of aortic valve replacement (AVR) or death than subjects with lower levels [45]. This appears to be mediated by the pro-osteogenic effects of Lp(a) and OxPL on valvular interstitial cells, and these effects are potentially reversible with targeted treatment inactivating OxPL [46,47]. 

A recent publication by Kaiser et al. showed that, in the population-based longitudinal Rotterdam Study with over 14 years of follow-up (n = 922), Lp(a) was robustly associated with baseline and new-onset aortic valve calcification but not with progression of calcification, suggesting that Lp(a)-lowering interventions may be most effective in the pre-calcific stages of aortic valve diseases [48].

### 3.4. Aortic Dissection

It is well-known that Lp(a) plays a role in promoting monocyte trafficking to arterial walls and that its OxPL content promotes an inflammatory response in the arterial wall and subsequent vascular injury [49]. Due to the sudden onset of AD, its high mortality, and poor prognosis, it is imperative that we identify the unidentified potential risk factors in order to intervene at an early stage. A recent study of 200 patients with AD and 200 controls showed that patients with AD had greater median Lp(a) concentrations than non-AD cases (152.50 vs. 81.75 mg/L) [34]. However, due to some study limitations and the lack of additional evidence about this potential association, future large cohort or Mendelian randomization studies are needed to determine the causal relationship between Lp(a) and AD.

### 3.5. Peripheral Arterial Disease

High Lp(a) levels and their association with PAD have also been demonstrated in several studies [50,51,52,53,54]. A study published by Klarin et al. identified new LPA loci that were associated with PAD, suggesting that the modulation of circulating Lp(a) may result in decreasing the risk of developing this condition [55]. An important aspect of PAD suggested by Golledge et al. in a recent publication is the relationship between serum Lp(a) concentrations and the requirement for PAD interventions [56]. In this research, the authors assessed 1472 patients with a follow-up of 2.4 years and showed that participants with Lp(a) ≥ 30 mg/dL had more extensive disease, resulting in a greater requirement for any PAD operation and lower-limb peripheral revascularization.

### 3.6. Heart Failure

Several studies have investigated the link between Lp(a) and HF. One study tested whether elevated Lp(a) levels and corresponding LPA risk genotypes (rs3798220 and rs10455872) were associated with an increased risk of HF. From a total of 98,097 patients, 4122 were diagnosed with HF. The authors concluded that elevated Lp(a) levels and corresponding LPA risk genotypes were associated with HF, increasing HF risk with higher Lp(a) percentile [57].

Agarwala et al. examined the association of Lp(a) levels with incident HF hospitalizations in the Atherosclerosis Risk in Communities (ARIC) study [58]. After a median follow-up of 23.4 years, individuals with higher levels of Lp(a) had an increased risk of HF hospitalization even after adjustment for other risk factors (age, race, gender, hypertension, DM, smoking status, body mass index, heart rate, and high-density lipoprotein cholesterol) [58].

### 3.7. Diabetes Mellitus

Several studies reported that type 1 DM patients have Lp(a) concentrations that are not different from healthy individuals if they are well-controlled and free from kidney dysfunction [59,60,61]. Type 2 DM patients, on the other hand, may have reduced Lp(a) levels due to mutations or polymorphisms in genes that affect the expression of the apo(a) gene and the phenotype of DM. 

In a study conducted by Kamstrup et al. with 80,000 patients, investigators measured plasma Lp(a) concentrations, the number of KIV-2 repeats, and the rs10455872 SNP [62]. They showed that type 2 DM patients had lower Lp(a) concentrations. Individuals with high numbers of KIV-2 repeats (that correlate with low plasma Lp(a) levels) showed a higher risk for type 2 DM. On the other hand, carriers of the rs10455872 SNP associated with elevated Lp(a) concentrations did not show a different risk of type 2 DM. The authors concluded that low Lp(a) concentrations by themselves might not be causal for increased type 2 DM risk, yet this might differ for individuals with a high number of KIV-2 repeats [63]. 

Moreover, work by Gudbjartsson et al. has shown similar findings; the 10% of subjects with very low Lp(a) concentration (<3.5 nmol/L) were reported to be at greater risk of type 2 DM. However, type 2 DM risk was independent of Lp(a) concentration in subjects with Lp(a) levels above the median (14 nmol/L) [64]. 

### 3.8. Thrombosis

Lp(a) has a pro-thrombotic potential by reducing PLG activation and fibrin degradation while increasing PLG Activator Inhibitor-1 expression on endothelial cells and the activity of the tissue factor pathway inhibitor. All of these mechanisms result in enhanced platelet activation and thrombus formation [29,65]. Elevated Lp(a) levels are correlated with reduced fibrin clot permeability and impaired fibrinolysis [33], which is more profound for smaller-sized isoforms of Lp(a) because these have higher affinity for fibrin [66]. Assessment of associations between genetically elevated Lp(a) levels and different forms of venous thrombosis, however, have demonstrated uniformly negative results, which rule out a role for elevated Lp(a) in the etiology of venous thrombosis [33,67,68]. In light of these findings, it is likely that venous thrombosis reflects impaired anti-coagulation/accelerated coagulation rather than impaired fibrinolysis and, likewise, that Lp(a) does not promote coagulation in vivo as much as it impairs fibrinolysis. 

## 4. Lp(a) Treatment

### 4.1. The Effect of Currently Available Therapies on Lp(a)

The effects of currently available lipid-modifying agents on Lp(a) are modest and variable, except for the monoclonal antibody PCSK9i that reported a potentially relevant reduction in Lp(a) levels [69,70].

The PSCK9i Lp(a)-lowering effects are related to the increase in Lp(a) clearance, resulting in a decrease in Lp(a) concentrations in the range of 14% to 35% [9,71]. Multiple secondary analyses from RCTs evaluating PCSK9i have provided evidence for their potential role in reducing Lp(a)-related cardiovascular risk [72,73,74,75]. Although the absolute reductions in Lp(a) to produce clinically significant lowering in CVD risk is not definitively established, the magnitude of the effect of these therapies seems insufficient to appropriately reduce Lp(a)-related CVD risk [76]. In a secondary analysis from the FOURIER (Further Outcomes Research With PCSK9 Inhibition in Subjects With Elevated Risk) study, median Lp(a) reduction with evolocumab was 26.9%, and patients with higher baseline Lp(a) levels (greater than 37 nmol/L) experienced greater reductions in Lp(a) and tended to show greater cardiovascular benefits from PCSK9i treatment [72]. Data derived from ODYSSEY OUTCOMES (*Evaluation of Cardiovascular Outcomes After an Acute Coronary Syndrome During Treatment With Alirocumab*) trials confirmed a significant median reduction in Lp(a) with alirocumab of 25.6%, along with a reduction in the risk of MACE [69]. However, controversy exists whether the Lp(a) reductions were significantly associated with a decrease in the risk of MACE independent of LDL reductions [77]. While some studies suggested that Lp(a) lowering by alirocumab was an independent contributor to MACE reduction [73,78], other publications demonstrated that, in patients with low Lp(a) levels (<13.7 mg/dL) and LDL at or near target levels, alirocumab did not further reduce the risk of recurrent MACE [77]. In summary, although there is a reduction in Lp(a) levels seen with PCSK9i therapy, these results come from post hoc analyses of RCTs in patients with overall low Lp(a) concentrations, and it is controversial if these reductions are adequate to enable a significant lowering of MACE. Regarding side effects, PSCK9i appears to be well tolerated; however, local injection site reactions (usually mild) are reported in 6–10% of evolocumab- and alirocumab-treated patients [71]. These drugs do not appear to cause severe muscle or liver toxicity.

There is substantial heterogeneity in the effects of statins on Lp(a) levels, ranging from a significant reduction demonstrated in the Collaborative Atorvastatin Diabetes Study (CARDS) to a suggested increase in the Scandinavian Simvastatin Survival Study (4S) [79,80]. In a subject-level meta-analysis including 5256 patients (1371 on placebo and 3885 on statin) from six RCTs, statins significantly increased plasma Lp(a) levels, with a mean percent change from baseline ranging from 8.5% to 19.6% [81]. Another meta-analysis using patient-level data from seven RCTs included 29,069 patients with repeated Lp(a) measurements. This study showed an independent approximately linear relation between Lp(a) levels and CVD risk, but the initiation of statin therapy had no significant effects on Lp(a) concentrations [6]. Muscle-related adverse reactions and hepatic dysfunction remain the main side effects related to statins’ use. Several RCTs have reported no significant increase in the incidence of persistently elevated aminotransferases, but a meta-analysis showed an excess risk of aminotransferases’ elevation of 0.4% [82]. A small increase in muscle symptoms was found in a recent meta-analysis; however, another meta-analysis conducted in 2014 found little or no excess risk of myalgias, creatine kinase elevations, or rhabdomyolysis when using a strict definition [83,84].

Cholesteryl Ester Transfer Protein (CETP) inhibition can reduce Lp(a) levels in the range of 25% to 40% [85,86]. In a placebo-controlled trial including 393 patients, Evacetrapib significantly reduced the concentrations of Lp(a) by 32% as monotherapy and by 31% when combined with a statin [86]. In a fixed-sequence, double-blind study of the effects of anacetrapib on the metabolism of apoB and high-density lipoproteins enrolling 39 patients, anacetrapib treatment lowered Lp(a) by 34.1% [85]. In a RCT including 120 dyslipidemic patients with background high-intensity statin treatment, obicetrapib significantly reduced Lp(a) levels [87]. However, CETP inhibitors did not demonstrate an overall benefit for MACE reduction and were mainly evaluated in trials that did not enroll patients based on elevated Lp(a) levels [69]. Therefore, drug development for most CETP inhibitors was halted because of futility or even adverse cardiovascular side effects [88]. The exception was anacetrapib that reduced CVD events on top of statin therapy and was not associated with major side effects. However, there was a small (0.7 mmHg) increase in systolic blood pressure in anacetrapib treated subjects, similar to that observed with evacetrapib and dalcetrapib, but less than for torcetrapib [88].

Niacin can also reduce Lp(a) levels in a range between 20 and 30%. In an RCT including 25,673 participants with ASCVD, niacin was associated with a reduction in Lp(a) levels but did not significantly reduce the risk of MACE [89]. Similarly, another RCT demonstrated that Lp(a) levels were associated with an increased CVD risk, but the use of niacin did not reduce the CVD risk despite favorable changes in Lp(a) [90]. Niacin was also associated with an increased risk of serious adverse events related to the gastrointestinal system, musculoskeletal system, skin, infection, and bleeding [89].

Lipoprotein apheresis, an intensive therapy that requires patients to attend several sessions, may be effective at lowering lower Lp(a) [69]. In a retrospective cohort study, lipoprotein apheresis demonstrated a reduction of 63% for Lp(a) and a 94% reduction in MACE over a mean treatment period of 48 months [91]. In a prospective observational multicenter study including 170 patients with Lp(a) hyperlipoproteinemia, progressive CVD, and maximally tolerated lipid-lowering medication, lipoprotein apheresis effectively lowered the incidence of MACE [92]. A phase-three prospective, multicenter, multinational, two-arm matched-pair cohort study whose primary objective is to demonstrate the clinical benefit of Lp(a) reduction using lipoprotein apheresis is ongoing [93]. Although data derived from apheresis studies appear promising, definitive results from RCTs of apheresis in the prevention or reduction of MACE are lacking. Moreover, this treatment may be not only cumbersome for patients but also implies the risk of adverse events, including vascular access complications and transient hemodynamic effects [94]. Lastly, this therapy is very infrequently used worldwide and may be only highly effective in the acute setting, but due to rapid hepatocyte synthesis, Lp(a) concentrations may return to baseline after 1 week [16].

### 4.2. Inclisiran

Inclisiran is a small interfering RNA (siRNA) that was shown to inhibit the hepatic synthesis of the PCSK9 protein [95]. It is the first-in-class siRNA against PCSK9 and has been recently approved by US Food and Drug Administration (FDA) and the European Medicines Agency (EMA) for the treatment of adults with heterozygous familial hypercholesterolemia or ASCVD who require additional LDL lowering despite diet and maximally tolerated statin therapy [96].

The effects of this agent on lipid molecules were tested in the ORION trials. ORION-1 was a phase-two trial assessing six different Inclisiran dosing regimens versus placebo and included 501 patients with elevated LDL levels despite maximally tolerated statin therapy [97]. In this study, Inclisiran reduced Lp(a) levels in the range of 14% to 26%. In the ORION-3 trial, a 4-year open-label extension study of the ORION-1 trial [97,98], Lp(a) levels were reduced by 6.3% in the Inclisiran-only arm and by 14.3% in the switching arm. The ORION-9 trial was a phase-three study that included 482 patients with heterozygous familial hypercholesterolemia receiving Inclisiran or placebo; in the Inclisiran group, the median levels of Lp(a) were reduced by 17.2% from baseline [99]. 

In two phase-three studies, patients with ASCVD (ORION-10 trial) and those with ASCVD or an ASCVD risk equivalent (ORION-11 trial) who had elevated LDL cholesterol levels despite statin therapy at the maximum tolerated dose were enrolled [100]. Inclisiran lowered levels of Lp(a) by 25.6% (ORION 10) and 18.6% (ORION 11), and drug-related adverse events were generally similar in the Inclisiran and placebo groups, although injection-site adverse events were more frequent with Inclisiran. In a pre-specified analysis of the ORION-11 trial that included 203 individuals at risk of, but without prior ASCVD, and LDL ≥ 2.6 mmol/L despite maximally tolerated statins, Inclisiran reduced Lp(a) levels by 28.5% [101].

The infrequent dosing regimen (twice yearly) offers a prolonged lipid-lowering effect, supporting a substantial and sustained lipid-lowering response among patients treated with Inclisiran [101]. This may help overcome non-adherence issues, which are a major cause of not achieving medical targets with existing therapies.

### 4.3. Novel Therapies

As already detailed, most lipid-modifying therapies do not have a substantial impact on the reduction of Lp(a)-related MACE. Therefore, with advancing technology, new therapies are emerging, including RNAi agents [8], which are promising in their potential to address Lp(a)-mediated CVD risk [69]. RNAis are a subtype of oligonucleotide therapeutics, and their mechanism of action is linked with the temporary and reversible downregulation of gene expression [69]. These pharmacological interventions are therefore very specific, and further precision has been achieved through the conjugation of RNAi to a cluster of amino sugars, the N-acetyl galactosamines (GalNAc) [102]. GalNAc is a ligand for the asialoglycoprotein receptors, which are found in large concentrations on the surface of hepatocytes with a high degree of cell-type specificity [22]. Conjugation of RNAi molecules to GalNAc clusters therefore directs these agents near-exclusively to hepatocytes, where their action can be targeted without affecting other unintended cell types [22].

Considering the Lp(a) structure and its production, two genes may be potential targets to reduce serum Lp(a) levels. However, APOB is not specific for Lp(a), and loss-of-function mutations in APOB can lead to hepatic steatosis [103]. In contrast, the LPA gene is highly specific for Lp(a), thus making this gene a more attractive aim for Lp(a) reduction. By specifically downregulating LPA in hepatocytes, the production and secretion of Lp(a) by the liver can be reduced.

**Figure 2:**
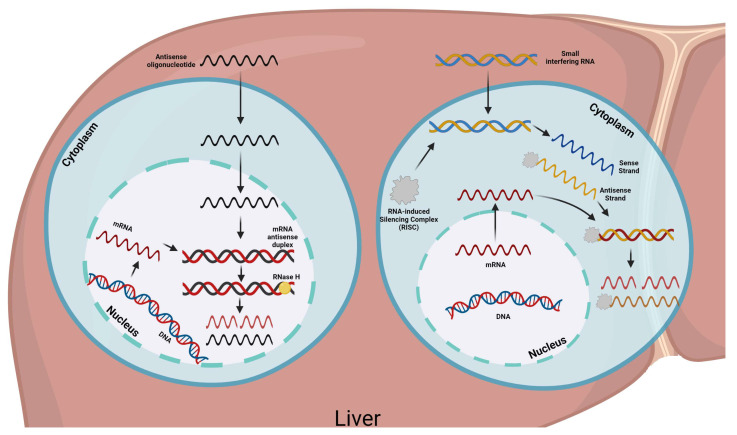
RNA interference agents include small interfering RNA (siRNA) and antisense oligonucleotides (ASOs). siRNAs are capable of blocking gene expression by interaction with mRNA before its translation. ASOs can alter RNA and reduce, restore, or modify protein expression through several distinct mechanisms. Novel treatments that inhibit the translation of mRNA for proteins specifically involved in lipid metabolism are currently being developed, and siRNAs and ASOs to lower Lp(a) are under study.

The most common RNAi agents include siRNA and antisense oligonucleotides (ASOs); siRNA consists of a class of drugs capable of blocking gene expression by interaction with mRNA before its translation and has the ability to silence the expression of disease-causing genes [104]. ASOs are short, synthetic, single-stranded oligodeoxynucleotides that can alter RNA and reduce, restore, or modify protein expression through several distinct mechanisms [105] (Figure 2). 

Different siRNAs and ASOs have demonstrated effectiveness in clinical studies against other therapeutic targets, such as the siRNA therapeutic agents Patisiran (targeting hereditary ATTR amyloidosis) and Givosiran (targeting acute hepatic porphyria) [106,107]. Novel treatments that inhibit the translation of mRNA for proteins specifically involved in lipid metabolism are currently being developed, and siRNAs and ASOs to lower Lp(a) are currently under study [108] (Table 1).

#### 4.3.1. Olpasiran

Olpasiran is a hepatocyte-targeted siRNA against the LPA gene that emerged as the best candidate for clinical testing from an initial group of 108 siRNAs specific for LPA that were selected using a bioinformatics algorithm [109]. This agent reduced Lp(a) concentrations in transgenic mice and cynomolgus monkeys in a dose-responsive manner, achieving up to over 80% reduction [109].

A phase-one clinical trial evaluating the safety, tolerability, pharmacokinetics, and pharmacodynamics of single doses of Olpasiran versus a placebo included patients with elevated plasma levels of Lp(a) who were grouped in different cohorts according to their Lp(a) concentrations (≥70 nmol/L and ≤199 nmol/L, or ≥200 nmol/L) [109]. Olpasiran reduced the mean Lp(a) levels at day 43 by 71% to 96% (in patients with Lp(a) levels between 70 and 199 nmol/L) and by 75% to 89% (in patients with Lp(a) concentrations higher than 200 nmol/L). No safety concerns were identified. Two phase-one studies are evaluating the pharmacokinetics, safety, and pharmacodynamics of this agent in patients with various degrees of hepatic impairment (NCT05481411) [110] and in participants with various degrees of renal dysfunction (NCT05489614) [111]. Both studies are currently recruiting participants.

A phase-two randomized, double-blind, placebo-controlled, dose-finding trial of this drug (Olpasiran Trials of Cardiovascular Events and Lipoprotein(a) Reduction (OCEAN[a]-DOSE) was recently reported [112]. This study included 281 patients with established CVD and Lp(a) concentrations higher than 150 nmol/L. Four doses of Olpasiran were tested, resulting in a significant reduction of Lp(a) concentrations in a dose-dependent manner [112]. The overall incidence of adverse events was similar across the Olpasiran and placebo groups, and the most common Olpasiran-related adverse events were injection-site reactions. 

A phase-three study with this agent is ongoing (Olpasiran Trials of Cardiovascular Events and Lipoprotein(a) Reduction (OCEAN(a))—Outcomes Trial). This double-blind, randomized, placebo-controlled multicenter study plans to include 6000 participants between 18 and 85 years old with elevated Lp(a) levels (≥200 nmol/L) and with a history of myocardial infarction and/or coronary revascularization [113].

#### 4.3.2. Pelacarsen

Pelacarsen is a second-generation ASO that is currently being tested in different trials. In a phase-two randomized, double-blind, placebo-controlled, dose-ranging trial involving 286 patients with established CVD and Lp(a) levels ≥60 mg/mL, Pelacarsen resulted in a dose-dependent significant decrease in Lp(a) levels, with mean percent decreases ranging from 35% at the lowest dose and 80% with the highest dose [60]. No significant differences between any Pelacarsen dose and placebo were seen with respect to serious side effects. 

Another phase 1/2a randomized, placebo-controlled study enrolled 58 participants with elevated Lp(a) concentrations. Participants assigned to Pelacarsen demonstrated mean Lp(a) reductions between 26% and 92%, suggesting that this drug may be a tolerable, potent therapy to reduce Lp(a) concentrations [114]. 

A phase-three randomized, double-blind, placebo-controlled multicenter study (NCT04023552, HORIZON trial) is ongoing, with the aim of supporting the indication of Pelacarsen for the reduction of cardiovascular risk in patients with established CVD and elevated Lp(a) [108]. This study randomizes patients with Lp(a) ≥ 70 mg/dL who are receiving optimal LDL-cholesterol-lowering treatment and who have a history of myocardial infarction, ischemic stroke, or clinically significant symptomatic PAD to receive Pelacarsen 80 mg injected monthly subcutaneously versus a placebo. The aim of this study is to evaluate the clinical-event-related impact of this new treatment on the incidence of cardiovascular death, non-fatal myocardial infarction, non-fatal stroke, or urgent coronary revascularization.

#### 4.3.3. SLN360

SLN360 is a chemically stabilized, double-stranded siRNA conjugated to GalNAc that interferes with the biosynthesis of Lp(a) in the liver [22]. It forms a complex with the transcription product of the LPA gene (LPA-mRNA) and facilitates its degradation, thus preventing translation and impeding the production of apo(a) [108]. SLN360 was tested in vitro, using human hepatocytes, and in vivo, using cynomolgus monkeys. These preclinical studies showed a potent reduction in LPA mRNA, while no effect was observed for the expression of APOB. In vivo, SLN360 administration resulted in a specific LPA mRNA reduction (up to 91%) and a potent (up to 95%) and long-lasting (≥9 weeks) serum Lp(a) reduction. The minimally effective dose was determined to be 0.3 mg/kg subcutaneously [22].

A phase-one randomized, single-ascending-dose clinical trial of SLN360 enrolled 32 adults with high Lp(a) plasma concentrations (≥150 nmol/L) and no known CVD [115]. In this study, SLN360 was well tolerated, and a dose-dependent lowering of serum Lp(a) levels was observed (46% to 98% reduction). An additional part of this study (NCT04606602) is recruiting patients with elevated plasma Lp(a) (≥150 nmol/L), a body mass index of ≥18 kg/m^2^ and ≤45 kg/m^2^, and a confirmed history of stable ASCVD. This study will primarily evaluate the incidence of treatment-emergent adverse events at day 150 [116].

The “Evaluate SLN360 in Participants with Elevated Lipoprotein(a) at High Risk of Atherosclerotic Cardiovascular Disease Events” (NCT05537571) is a phase-two ongoing study that includes patients with Lp(a) ≥ 125 nmol/L who are at high risk of ASCVD events and who have a body mass index in the range of 18.0 to 32.0 kg/m^2^ [117]. The primary aim of this study is to determine the safety and efficacy of this siRNA in larger populations and establish the time-averaged change in Lp(a) from baseline to week 36.

#### 4.3.4. LY3819469

Lastly, LY3819469, a GalNAc-conjugated siRNA, is under study for the treatment of elevated Lp(a). A phase-one study (“A Study of LY3819469 in Healthy Participants”, NCT04914546) to evaluate the safety and tolerability of LY3819469 in healthy participants with high Lp(a) levels is completed, but the results are not yet available [118]. A phase-two study is planned but yet to commence. The goal is to recruit 254 participants of at least 40 years of age, with Lp(a) ≥ 175 nmol/L and on a stable regimen for at least 4 weeks prior to screening [119]. The primary outcome will be the percent change from baseline in time-averaged Lp(a).

## 5. Future Perspectives

Genetic data, including LPA genotype, are becoming more readily available, and they may provide valuable information to supplement Lp(a) serum concentrations’ measurements in the assessment of CVD risk [69]. Additionally, genetic information could help evaluate pharmacogenetic interactions of RNAi therapeutics and identify likely responder patients [69]. As a limitation, genetic information regarding SNPs in the LPA gene is predominantly limited to European populations, while it is known that Lp(a) concentrations in various ethnicities are less dependent on LPA SNPs alone [120,121]. The complexity of the genetic variants in the LPA gene remains an area that needs further exploration, and the impact of ethnicity on Lp(a) levels should be considered in future studies.

HORIZON (Pelacarsen) and OCEAN(a) (Olpasiran) phase-three trials will establish the populations with the largest benefit from Lp(a)-lowering therapies in secondary prevention. Following the completion of these studies, Lp(a)-lowering therapeutics will likely be evaluated in studies for primary prevention. In the primary prevention trials, inclusion criteria such as LDL levels before enrollment, baseline maximally tolerated LDL-lowering therapy, inflammatory markers, and, most importantly, thresholds for elevated Lp(a) should be accurately addressed [9]. Regarding this last point, data from trials and Mendelian randomization analyses have suggested a cutoff value of 175 nmol/L (70 mg/dL) to be sufficient to reach the necessary Lp(a) reduction to achieve significant benefits in the prevention of CVD outcomes [9]. However, this cutoff point should be considered a dynamic value, and the demographic characteristics of the included population should be taken into account for its definition.

Furthermore, analyses of the results according to ethnicity will help determine thresholds for potential ethnicity-focused trials. Lastly, imaging endpoints helped demonstrate the efficacy of other lipid-lowering therapies on atherosclerosis; thus, these could also be utilized in Lp(a) therapies trials as surrogate endpoints to evaluate subclinical atherosclerosis [122].

## 6. Summary and Conclusions

Lp(a) is primarily genetically determined, and elevated levels are strongly associated with the development of CVD and clinical events. It is a laboratory measure that is widely accessible. However, until recently, it was not considered possible to effectively treat it in clinical practice for the prevention of CVD, with limited impact afforded by statins. Meanwhile, PCSK9i therapy did not demonstrate an independent effect on Lp(a)-related CVD outcomes despite lowering Lp(a) levels. Novel therapies aimed at clinically significant Lp(a) reduction include RNA interference agents, which have the capacity for temporary and reversible downregulation of gene expression, with numerous agents now under investigation. These emerging therapies that are designed to specifically lower Lp(a) levels with a directly related reduction in attributable CVD outcomes hold the promise of a new era in CVD prevention for patients with a genetically mediated higher risk (Graphical Abstract).

## Figures and Tables

**Table 1:** Main completed and ongoing trials related to new Lp(a)-lowering therapies. RNAi: RNA interference. GalNAc–siRNA: N-acetylgalactosamine-conjugated small interfering RNA. Lp(a): lipoprotein(a). ASO: antisense oligonucleotides.

Agent	Phase of Development	Study Name	Efficacy Data	Safety Data
Olpasiran (GalNAc–siRNA)	Phase 3 (Ongoing)	OCEAN(a)—Outcomes NCT05581303	Not yet available. Inclusion Criteria Elevated Lp(a) levels (≥200 nmol/L) with a history of either a myocardial infarction and/or coronary revascularization. Primary Outcome Time to coronary heart disease death, myocardial infarction, or urgent coronary revascularization.	Not yet available.
	Phase 2 (Completed)	OCEAN(a)-DOSE NCT04270760	At 36 weeks, Olpasiran significantly reduced the Lp(a) concentrations in a dose-dependent manner (from 70% to 100%).	The incidence of adverse events was similar across all the groups. The most common adverse events were injection-site reactions.
	Phase 1 (Completed)	“Safety, Tolerability, Pharmacokinetics and Pharmacodynamics Study of AMG 890 in Subjects with Elevated Plasma Lipoprotein(a)” NCT03626662	Olpasiran reduced Lp(a) levels by 71% to 96% (in patients with Lp(a) levels between 70 and 199 nmol/L) and by 75% to 89% (in patients with Lp(a) concentrations higher than 200 nmol/L).	No serious safety concerns were identified.
	Phase 1 (Completed)	“A Study to Evaluate the Pharmacokinetics, Pharmacodynamics, Safety and Tolerability of Olpasiran in Chinese Participants with Elevated Serum Lipoprotein(a)” NCT04987320	The maximal Lp(a) reduction occurred at day 57, with mean Lp(a) reductions ranging from 56% to 99%	Adverse events were mild in severity, with no serious or fatal adverse events. No relevant changes in tolerability-related laboratory analytes or vital signs were observed.
	Phase 1 (Ongoing)	“A Study to Evaluate the Pharmacokinetics, Safety, and Pharmacodynamics of Olpasiran in Participants with Various Degrees of Hepatic Impairment” NCT05481411	Not yet available.	Not yet available.
	Phase 1 (Ongoing)	“A Study to Evaluate the Pharmacokinetics, Safety, and Pharmacodynamics of Olpasiran in Participants with Normal Renal Function and Participants with Various Degrees of Renal Impairment” NCT05489614	Not yet available.	Not yet available.
Pelacarsen (ASO)	Phase 3 (Ongoing)	HORIZON Trial NCT04023552	Not yet available. Inclusion Criteria Lp(a) ≥ 70 mg/dL and a history of myocardial infarction, ischemic stroke, or clinically significant symptomatic peripheral artery disease. Primary outcome Time to first occurrence of major adverse cardiovascular events.	Not yet available.
	Phase 3 (Ongoing)	“A Multicenter Trial Assessing the Impact of Lipoprotein(a) Lowering with Pelacarsen (TQJ230) on the Rate of Weekly Lipoprotein Apheresis Sessions in Patients with Hyperlipoproteinemia(a) and Established Cardiovascular Disease in Germany” NCT05305664	Not yet available. Inclusion Criteria Patients undergoing lipoprotein apheresis for isolated Lp(a) with Lp(a) >60 mg/dL and prior myocardial infarction, ischemic stroke, and/or clinically significant symptomatic peripheral artery disease. Primary outcome Rate of lipoprotein apheresis sessions performed over 52 weeks normalized to the weekly lipoprotein apheresis schedule.	
	Phase 2 (Completed)	“Phase 2 Study of ISIS 681257 (AKCEA-APO(a)-LRx) in Participants with Hyperlipoproteinemia(a) and Cardiovascular Disease” NCT03070782	Pelacarsen resulted in dose-dependent decreases in lp(a) levels from 35% to 80%.	Injection-site reactions were the most frequently reported adverse event and occurred in 27% of the patients who received Pelacarsen.
	Phase 1/2a (Completed)	“Safety, Tolerability, Pharmacokinetics, and Pharmacodynamics of IONIS APO(a)-LRx in Healthy Volunteers with Elevated Lipoprotein(a)” NCT02414594	Significant dose-dependent reductions in mean Lp(a) concentrations were noted in patients treated with Pelacarsen (single dose 26% to 85%; multiple doses 66% to 92%).	No significant differences between any Pelacarsen dose and placebo were seen with respect to side effects.
	Phase 1 (Completed)	“A Study to Assess Safety, Tolerability, Pharmacokinetics, and Pharmacodynamics of Pelacarsen (ISIS 681257) in Healthy Japanese Participants” NCT05337878	Not available.	Not available.
	Phase 1 (Completed)	“Study to Assess the Pharmacokinetics of Pelacarsen (TQJ230) in Participants with Mild Hepatic Impairment Compared to Matched Healthy Participants” NCT05026996	Not available.	Not available.
SLN360 (GalNAc–siRNA)	Phase 2 (Active, not recruiting)	“Evaluate SLN360 in Participants with Elevated Lipoprotein(a) at High Risk of Atherosclerotic Cardiovascular Disease Events” NCT05537571	Not yet available.	Not yet available.
	Phase 1 (Completed)	“Study to Investigate Safety, Tolerability, PK and PD Response of SLN360 in Subjects with Elevated Lipoprotein(a)” NCT04606602	Significant dose-dependent lowering of plasma Lp(a) concentrations was observed with SLN360 treatment (from 46% to 98%).	No significant side effects. One participant experienced two serious adverse event episodes judged to be unrelated to study drug.
LY3819469 (GalNAc–siRNA)	Phase 1 (Completed)	“A Study of LY3819469 in Healthy Participants” NCT04914546	Not available.	Not available.
	Phase 2 (Active, not recruiting)	“A Study of LY3819469 in Participants with Elevated Lipoprotein(a) [Lp(a)]” NCT05565742	Not yet available.	Not yet available.

## Data Availability

Data is contained within the article.
